# Editorial: Orchid genomics and developmental biology, volume II

**DOI:** 10.3389/fpls.2023.1136350

**Published:** 2023-01-16

**Authors:** Katharina Nargar, Jen-Tsung Chen

**Affiliations:** ^1^ Australian Tropical Herbarium, James Cook University, Cairns, QLD, Australia; ^2^ National Research Collections Australia, Commonwealth Industrial and Scientific Research Organisation (CSIRO), Canberra, ACT, Australia; ^3^ Department of Life Sciences, National University of Kaohsiung, Kaohsiung, Taiwan

**Keywords:** orchid, biotechnology, functional genomics, developmental biology, omics

Orchidaceae constitute the second-largest flowering plant family worldwide with over 27,000 species found on all continents except Antarctica. Orchids are frequently celebrated for their exceptional morphological and ecological diversity and are highly valued in the horticultural trade. Orchids exhibit distinct floral and physiological features, such as fused male and female flower parts forming the gynostemium, a floral lip often adorned with calli, glands, spurs, and distinctive color patterns, and the crassulacean acid metabolism (CAM), a water-saving physiological pathway which has evolved multiple times independently within the family. Orchids possess highly specialized ecological relationships, such as often species-specific plant-pollinator interactions including food- and sexual deception and dependence on mycorrhizal fungi for germination of their minute seed. The broad range of traits renders orchids prime non-model plants for elucidating the genomic underpinnings and regulatory networks responsible for the generation of this exceptional diversity. To keep exploring such an interesting research field, this volume continues our previous Research Topic entitled “Orchid Genomics and Developmental Biology” **(** Chen and Nargar **)** to showcase recent findings and providing novel insights into regulatory mechanisms underpinning orchid biologies, such as reproductive development, responses to biotic and abiotic stresses, visual mimicry of orchid flowers, and bioactive metabolic pathways.

## Regulatory mechanisms underpinning reproductive development in orchids

Ovule development plays a vital role in plant reproduction and seed development. However, the regulatory mechanism underpinning ovule development in orchids is poorly understood. Zeng et al. present a comparative transcriptomic and metabolomic study of ovules of different developmental stages in *Cymbidium sinense*. Among 9845 differentially expressed unigenes (DEUs), the team identified candidate genes involved in ovule development, such as homeobox and MADS-box transcription factors, and phytohormones, such as cytokinin, gibberellin, and abscisic acid.

*SEPPALATA*-like MADS-box genes encode transcription factors which are responsible for floral organ specification. Cheng et al. investigated the role of *SEPPALATA*-like (*SEP*-like) MADS-box genes in the flower development of venus slipper orchids (*Paphiopedilum*). The team identified three *SEP*-like genes with phylogenetic placement in the core eudicot *SEP3* lineage. Protein-protein interactions indicated that the *SEP*-like genes may interact with B-class and E-class proteins, providing further insights into the role of *SEP*-like genes in the floral development of orchids.

Genes encoding for the YABBY transcription factor family play significant roles in lateral organ development, such as cotyledons and floral organs. Based on genomic sequence data, Wang et al. identified and characterized 24 *YABBY* genes in three ornamental *Cymbidium* species. Expression analysis showed that *YAB2* genes were expressed more strongly in floral organs than in vegetative tissue. The authors identified two *YABBY* genes which were mainly expressed in the gynostemium.

The gynostemium is a remarkably complex structure of fused female and male organs. However, the multi-level regulatory networks in its formation are largely unknown. Yang et al. examined the role of microRNA in flower formation in *Cymbidium ensifolium*. The team found that a distinct microRNA (*Ce*-miRNA396) silenced Growth-Regulating Factors (GRFs) through cleavage. The team showed that GRF transcripts accumulated most in floral tissues where the miR969 concentration was lowest, and found a strong correlation between *Ce*-miRNA396, floral formation, and column specification.

Many orchids of horticultural interest require several years from propagation *via* seeds to flowering, hence shortening this time span is a key desideratum. Ahmad et al. investigated the regulatory mechanism of the curious phenomenon of flower formation in protocorms without prior formation of leaves or roots in three *Cymbidium* species. Through comparative transcriptome analysis, the team identified transcription factor (TF) families and candidate key TFs involved in the regulatory networks that govern the onset of the reproductive stage.

## Underpinnings of visual mimicry in sexually-deceptive orchids

Pollination by sexual deception has evolved multiple times independently in Orchidaceae, with floral color playing a key role in visual mimicry. Wong et al. investigated the chemical composition and genetic regulatory networks underpinning floral coloration in a sexually deceptive bird orchid (*Chiloglottis trapeziformis*). The study elucidated the complex tissue-specific regulation of genes and biochemical pathways, in particular of the anthocyanin and flavonol glycoside metabolic pathways, across different stages of flower development.

In a related study, Wong et al. examined the chemical and genetic basis for floral coloration across the genus *Chiloglottis.* Phylogenomic analysis resolved three main evolutionary lineages, with the Formicifera clade sister to the Reflexa clade, and the two in turn sister to the Valida clade. While the biochemical basis of the distinct flower coloration underlying the floral mimicry was found to be conserved within the genus, biochemistry and gene expression levels were more similar among the two more closely related Formicifera and Reflexa lineages compared to the more distantly related Valida clade.

## Regulatory mechanisms of bioactive metabolic pathways in orchids

Orchids used in traditional medicine are valued for their bioactive compounds. To alleviate anthropogenic pressure on wild orchids, *in vitro* culture, such as plant tissue culture, is increasingly established. To facilitate the selective enhancement of bioactive metabolites through *in vitro* systems, knowledge about the regulatory mechanism in the biosynthetic pathways is key. Bhattacharyya et al. investigated the metabolic pathways involved in the biosynthesis of key secondary metabolites in *Malaxis acuminata*, a threatened orchid valued in traditional medicine. The study provided insights into the regulatory pathways for the phytosterol β-sitosterol and the phenylpropanoids eugenol and isoeugenol. The study identified leaves as a potential alternate source for the production of bioactive metabolites to facilitate the sustainable use of this threatened species.

In another study on bioactive compounds in orchids, Ahmad et al. investigated the regulatory pathways of flavonoids and bibenzyls in *Arundina graminifolia*. The team identified candidate genes involved in the biosynthesis pathways of these bioactive compounds, including *BIBSY212*, *CYP84A1*, *CYP73A4*, *4CLL7*, *UGT88B1*, *UGT73C3*, *ANS*, *PAL*, *FLS*, and *CHS8*. Most of the candidate genes were expressed highest in leaves and roots. The concentration of phenylpropanoids was found to be highest in leaves, flavonoids in stems, and bibenzyl in leaves.

## Mechanisms underlying abiotic stress responses in epiphytic orchids

Zhang et al. investigated salinity stress responses in the epiphytic orchid and facultative CAM plant, *Dendrobium officinale*. The team showed that plants exposed to different levels of salinity stress in their roots responded with changes in the expression of genes related to hormone biosynthesis and response, amino acid and flavonoid metabolism, and the Salt Overly Sensitive (SOS) pathway. Key candidate genes playing a role in salt stress response in *D. officinale* were identified.

To investigate the morphological plasticity of aerial roots and associated regulatory networks in epiphytic orchids, Tian et al. undertook a detailed study of roots under terrestrial, epiphytic, and lithophytic growth conditions and different auxin treatments in *Dendrobium officinale* and *D. catenatum*. Differential expression analysis identified genes associated with the promotion of root elongation growth, which included upregulated auxin transporters and cellulose synthetase genes under low auxin levels. Genes associated with cell proliferation under high auxin levels included transportation and signal transduction pathways and stem cell control and regeneration pathway-related genes. WUSCHEL-related homeobox transcription factor WOX12 from *D. catenatum* was found to confer highly efficient pluripotency acquisition properties, relevant to monocot plant transformation such as in orchids.

Transcription factors of the WRKY family play important roles in plant responses to biotic and abiotic stresses and secondary metabolism. Wei et al. studied WRKY transcription factors in *Cymbidium sinensis*. Among 64 *WRKY* genes, the team identified key candidate genes, in particular in GROUP III, which were strongly induced in response to hormone treatments, indicating their potentially essential role in hormone signaling. The transcription factor CsWRKY18 was found to be associated with increasing plant tolerance to abiotic stress within the abscisic-acid- (ABA) dependent pathway.

## Novel properties in transposable elements in orchids

Transposable elements (TE) can have profound impacts on the host’s genomes, e.g., through increasing genome size. During their evolution, TE superfamilies have evolved significant changes in their architecture which can have profound impacts on their interaction with host organisms. Alvarado-Marchena et al. mined angiosperm genomes for new conserved protein domains within long terminal repeat (LTR) retrotransposons. The team discovered an additional open reading frame in Gypsy-type elements in *Phalaenopsis* orchids with similar properties to m6A RNA demethylase in AlkB proteins. The study demonstrated its RNA binding capacity and demethylase activity which may convey LTR retrotransposons increased fitness.

## Repurposing genomic resources in orchids

Transcriptome data for non-model organisms such as orchids has seen a massive increase and presents a valuable data resource. Wong and Peakall explored the use of multi-tissue transcriptomes to infer phylogenetic relationships in Orchidaceae at both deep and shallow evolutionary scales. The phylotranscriptomic approach yielded largely consistent findings with other phylogenomic studies. For the phylogenetic placement of mycoheterotrophic species, which can undergo severe plastome degeneration, phylotranscriptomics yielded results consistent with large-scale nuclear phylogenomic studies. The authors discuss other potential uses of genomic resources, including the mining of genomes and transcriptomes for single-copy gene sets of bespoke target capture bait kits.

## Author contributions

KN and J-TC drafted the manuscript. Both authors contributed to the article and approved the submitted version.

